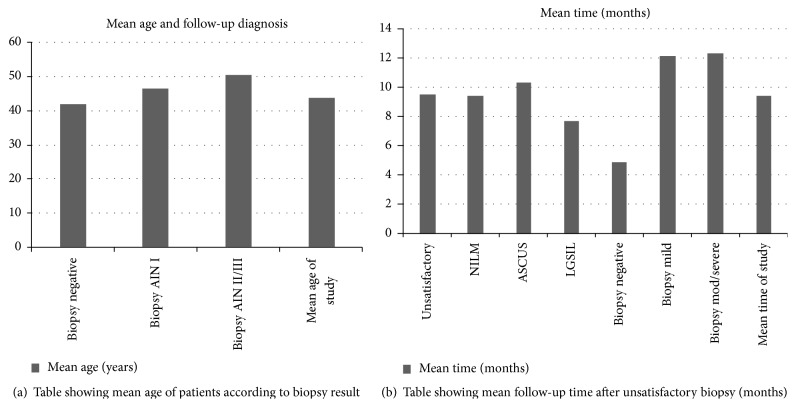# Histopathologic and Cytologic Follow-Up in High Risk Male Patients with Unsatisfactory Anal Cytology

**DOI:** 10.1155/2017/9780213

**Published:** 2017-09-18

**Authors:** Daniel J. Zaccarini, Kamal K. Khurana

**Affiliations:** Department of Pathology, SUNY Upstate Medical University, Syracuse, NY, USA

## Abstract

**Objective:**

Anal cytology is being increasingly used as part of anal cancer screening in patients at high risk for anal neoplasia. Most studies in anal cytology have focused on correlating the abnormal anal Pap smear with histopathologic outcomes. The aim of this study was to document histopathologic or repeat anal cytology outcomes in patients with unsatisfactory cytology.

**Materials and Methods:**

Unsatisfactory anal Pap tests in high risk male patients were correlated with follow-up histopathologic diagnoses or cytology.

**Results:**

1205 anal tests were performed during the study period and 214 (17.8%) were unsatisfactory. Adequate follow-up cytology was available in 75 cases and revealed epithelial cell abnormality (ECA) in 40% [30/75] (atypical squamous cells of undetermined significance (ASCUS) [20%] and low-grade squamous intraepithelial lesions (LGSIL) [20%]) and was negative for intraepithelial lesion or malignancy (NILM) in 60% [45/75] of cases. 28.7% of unsatisfactory Pap smears had unsatisfactory repeat cytology. Histopathological follow-up on these unsatisfactory Pap smears revealed anal intraepithelial neoplasia (AIN) 1 and AIN 2/3 or 2/3+ in 39% and 18% of the total number of biopsy cases, respectively.

**Conclusions:**

High risk male patients with unsatisfactory Pap smears are at significant risk of epithelial cell abnormality and histopathologically verifiable anal intraepithelial lesions.

## 1. Introduction

Anal cytology is an important preventative screening method for patients at risk for anal carcinoma. Patients at risk include men who have sex with men (MSM), HIV-positive men and women, women with a history of lower genital tract neoplasms, and transplant recipients [1]. Anal cancer is not a common cancer; it is the 26th most common cancer in the United States and represents only 0.4% of all new cancer cases [2]. However, in people living with HIV/AIDS it is the fourth most common cancer. The incidence of anal cancer in HIV-infected MSM is estimated at 131 per 100,000 person-years and surpasses the rate of cervical cancer in women prior to the initiation of screening [3, 4]. The incidence of AIN has not decreased since highly active antiretroviral therapy (HAART) therapy began [5]. Survival has increased in HIV-positive patients due to HAART and this has contributed to the increased risk of subsequently developing anal cancer in this patient population since patients are less likely to die from HIV-related complications [6].

**Figure 1:**
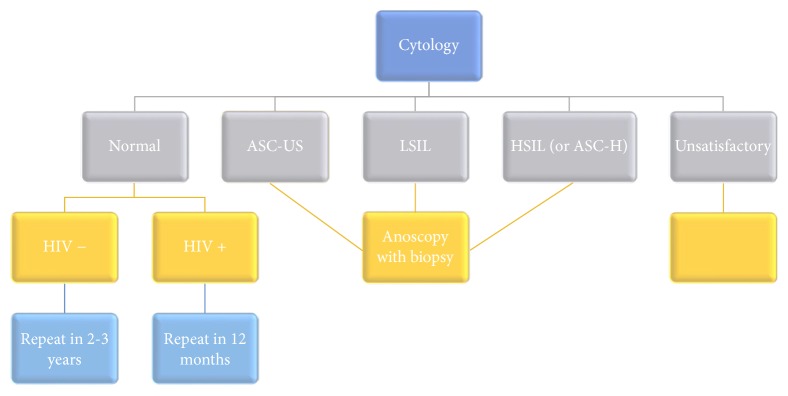
Recommendations for follow-up of anal cytology diagnoses.

Anal cytology is categorized according to the Bethesda system for cervical cytology [7]. Currently, no standard screening program for anal cancer screening exists, although recommendations have been proposed for general screening (Figure 1). Patients with normal cytology are recommended to have a repeat Pap smear in 12 months if HIV is positive or two to three years if HIV is negative [7]. Those with atypical squamous cells of undetermined significance (ASC-US), low-grade squamous intraepithelial lesion (LSIL), high-grade squamous intraepithelial lesion (HSIL), or atypical squamous cells which cannot rule out HSIL (ASC-H) are recommended for anoscopy [7]. However, the appropriate time interval for future screening in unsatisfactory anal Pap smears is not established.

Most studies in anal cytology have focused on correlating the abnormal anal Pap smear with histopathologic outcome and have not provided any follow-up on unsatisfactory anal Pap smears. Cytology often underestimates the grade of ASIL compared with the corresponding biopsy, although the positive predictive value of HSIL on anal cytology is very high [8]. In one study, comparing the results of anal cytology with biopsy, more than one-third of specimens with low-grade squamous intraepithelial lesions (LSIL) on anal cytology showed HSIL on biopsy [9]. The predictive value of cytology can be enhanced through repetitive testing and in one study, a repeat test after two years improved the positive predictive value of cytology from 38% to 78%, and the negative predictive value improved from 46% to 79% [10]. Studies on unsatisfactory follow-up have not been investigated. The aim of our study was to document histopathologic or repeat anal cytology outcomes in patients with unsatisfactory anal cytology.

## 2. Methods

After obtaining institutional review board exemption, a retrospective study was initiated. A computer-based search of the Copath laboratory information system database was carried out to retrieve all unsatisfactory anal Pap test results from January 2008 to December 2013. Follow-up clinical information, anal cytology, and histopathologic results were obtained from the Copath system. The proportion of ECA and SIL in patients with initial satisfactory anal Pap smears was also determined. All anal cytology samples were collected in Thin Prep PreservCyt Vial and were processed using ThinPrep Processor T2000 processor (Hologic, Marlborough, MA). This research was accepted as an abstract at the annual scientific meeting for the American Society of Cytopathology [11].

Staining of the slides was performed on a Leica ST5010 Autostainer XL (Leica Biosystems, Buffalo Grove, IL). All anal cytologic test slides were screened manually by cytotechnologists and then referred for additional pathologist review and signed out. Anal cytologic tests were reported using the 2001 Bethesda System criteria. As per TBS 2001, anal cytologic tests were judged as adequate for evaluation when 2,000 to 3,000 nucleated squamous cells were present. Criteria for an unsatisfactory Pap smear included samples predominantly composed of anucleate squames, samples obscured by fecal material, or due to sparse cellularity.

Student's *T*-test was performed to compare age of patients and time of follow-up for different cytologic and histologic diagnoses. The characteristics of the two cohorts, unsatisfactory and satisfactory cases during the study period, were compared using a chi-square test Office Excel 2016 (Microsoft, Redmond, and Wash). In all tests, a *p* value of ≤0.05 was considered a significant difference between the two compared sets of data.

## 3. Results

Out of the 1205 anal Pap smears performed during 2008 to 2013, 214 were unsatisfactory. Eleven females were excluded, and 60 males were excluded due to lack of follow-up, leaving 143 cases. All patients were HIV seropositive.

**Figure 2:**
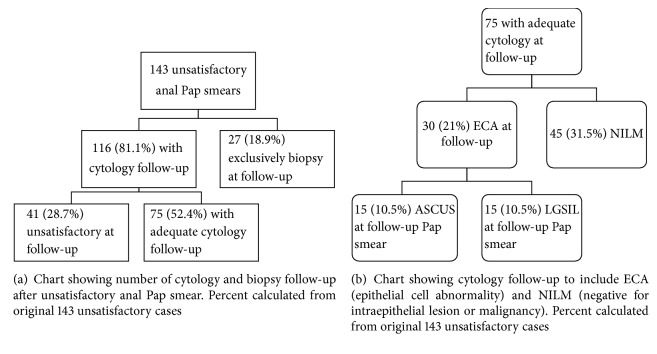


Of the 143 unsatisfactory anal Pap smears, 116 (81.1%) had cytology follow-up while 27 (18.9%) cases had exclusive biopsy follow-up (Figure 2(a)). Cytology follow-up continued to be unsatisfactory in 41 (28.7% of 143 cases) cases. Remaining 75 cases had adequate cytology follow-up comprised of 45 cases with NILM and 30 cases with ECA (Figure 2(b)). Of the 30 ECA (21% of 143 cases), 15 (10.5%) had ASCUS, and 15 had LGSIL (10.5% of 143 cases).

**Figure 3:**
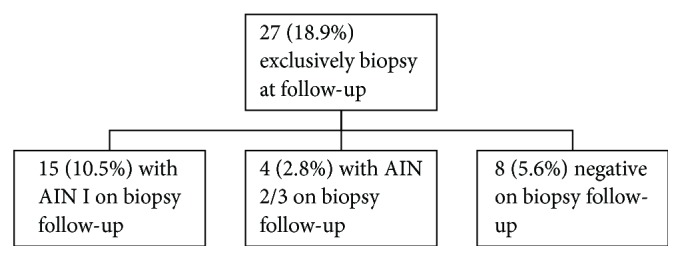
Chart showing exclusively biopsy follow-up results.

**Figure 4:**
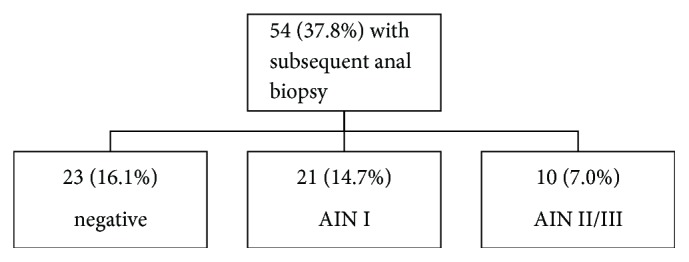
Chart showing outcome of all patients with biopsy follow-up after initial diagnosis of unsatisfactory anal Pap smear.

Fifteen (10.5%) AIN I and 4 (2.8%) AIN II/III cases occurred in the exclusively biopsy group (Figure 3) comprised of 27 cases. Reviewing all cases with biopsy follow-up (Figure 4) in the study showed 23 cases (16.1% of the original 143 unsatisfactory cases) that were negative and 31 (22%) cases with AIN (14.7% with AIN I and (7.0%) with AIN 2/3).

**Figure 5:**
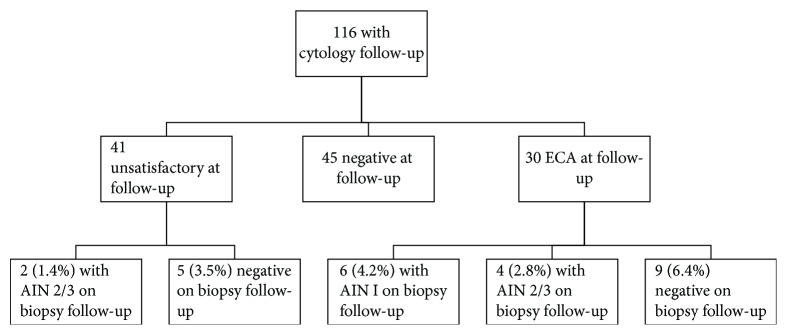
Chart showing all patients with subsequent anal biopsy results that had repeat anal cytology after an initial unsatisfactory anal Pap smear.

Figure 5 summarizes the histologic follow-up on 116 cases of unsatisfactory anal Pap smears with cytologic follow-up. Biopsy follow-up on these cases with repeat cytology revealed two high-grade AIN (1.4% of initial 143 unsatisfactory Pap smears) after repeat unsatisfactory cytology follow-up. Six cases of AIN I (4.2%) and four cases of AIN 2/3 (2.3%) were seen after ECA.

The mean ages of patients with subsequent negative biopsy were 41.9 years, while those with biopsy proven AIN2/3 or 2/3+ were higher (50.5 years). When comparing the age of patients or time of follow-up for different cytologic and histologic diagnoses after unsatisfactory cytology there was no statistically significant difference between the groups (*p* > 0.05) (Figures 6(a) and 6(b)). Follow-up time ranged from less than one month to up to 36 months. The mean age of the study was 43.9 years, with a range from 22 to 69 years.

There was no significant difference between the proportion of ECA in the unsatisfactory follow-up cohort with adequate cytology follow-up 40% (30/75) and the proportion of ECA cases with initial satisfactory anal Pap smears 44% (433/991) during the study time period (*p* > 0.05). The proportion of SIL in both groups was also not statistically significant (20% [15/75] versus 26.3% [263/991]).

## 4. Discussion

Anal-rectal cytology is considered a cost-effective screening tool for evaluating human papillomavirus-related disease of the anal canal, especially in at-risk populations, principally MSM and those with HIV disease [12]. Anal cytology is known to underestimate the grade of AIN on biopsy; however follow-up of unsatisfactory anal Pap smears has not been extensively studied [3, 10]. Unsatisfactory anal cytology rate of 17.8% in our study is similar to that reported by Morency et al. [13]. We also showed that 22% of the original 143 patients with unsatisfactory anal Pap smears showed an anal intraepithelial lesion (14.7% AIN I and 7% with AIN 2/3). Based on biopsy follow-up of 38 unsatisfactory anal Pap smears, Morency et al. reported 32% negative and 68% squamous intraepithelial lesions with 10.5% being of high grade [13]. In our follow-up of 54 unsatisfactory anal Pap cases with biopsy follow-up, distribution of squamous intraepithelial lesions (57.4%), HSIL (19%), and negative cases was not statistically significant when compared with that of Morency et al. (*p* = 0.185, chi-square test). Adequate cytologic follow-up of unsatisfactory cases (75) in our study revealed epithelial cell abnormality (ECA) in 40% (atypical squamous cells of undetermined significance (ASCUS) [20%] and low-grade squamous intraepithelial lesions (LGSIL) [20%]) and was negative for intraepithelial lesion or malignancy (NILM) in 60% of cases. We did not notice any significant difference in the proportion of cases with ECA and SIL in group with adequate anal cytology and unsatisfactory anal cytology cohort with cytologic follow-up.

Human papillomavirus (HPV) and HIV status are important considerations in patient cancer risk as well. It is unclear how immune function affects the risk of cancer. One review found that the prevalence of HPV infection in MSM and HIV-infected individuals is high, 86 to 98%, and HPV testing may not be useful [14]. The role of HPV testing in anal cytology remains to be elucidated and was not analyzed in this study.

There is a high likelihood of regression of HGSIL at two-year follow-up with only about 1-2% progressing to anal cancer [15]. Patients on antiretroviral therapy, who had low HIV viral load, and a CD4 count greater than or equal to 350/mm^3^ were not as likely to progress from lower grade lesions to higher grade lesions [15]. The role of viral load, medication status, and CD4 count was not assessed, and there is one limitation in this study. Studies have also demonstrated that HIV-positive patients with AIN I were more likely to progress to higher grade lesions, while HIV-negative patients were more likely to have no disease at two-year follow-up [16]. Despite these findings, AIN II/III is less likely to regress regardless of HIV status [17].

The American Society for Colposcopy and Cervical Pathology (ASCCP) approaches unsatisfactory Pap smears by stratifying patients by age and HPV status. Those that are HPV unknown or HPV negative and of age greater than 30 will receive a repeat cytology after two to four months [1]. Those greater than 30 years old who are HPV positive can get either repeat cytology in two to four months or colposcopy [1]. If repeat cytology is negative, then routine screening can be restarted. After two unsatisfactory Pap smears, colposcopy is recommended [1]. It is unclear whether HPV testing will be of any benefit in screening for anal cancer considering the high prevalence of HPV in this population and the other risk factors. Although similar guidelines for anal Pap smears have not been recommended, recent study by Naous et al. suggests that there may be need for age based screening in HIV-infected MSM population [18].

The abnormal cytology rate in anal Pap smears is much higher as compared to cervical Pap smears [19]. This may be related to the large percentage of patients in the former category that are HIV positive. The anal cytology abnormal rate among HIV-positive patients was 74.0% as compared to 52.5% in HIV-negative patients in one study [19]. Other factors related to the unsatisfactory rate include collection methods, experience with collection, and refraining from anal sex, douching, and taking an enema [5].

Limitations of the study included the small sample size and follow-up time. The risk of having an anal lesion in this study population is high. 28.0% (60 patients) of the original 214 unsatisfactory cases were lost to follow-up. This demonstrates that a significant number of intraepithelial lesions may be missed, and the importance of having a diagnostic sample is amplified.

In conclusion, high risk male patients with unsatisfactory Pap smears are at significant risk of epithelial cell abnormality and histopathologically verifiable anal intraepithelial lesions. Education related to better collection methods of anal Pap smears is required to reduce the unsatisfactory rate.

## Figures and Tables

**Figure 6:**